# ICT-based system to predict and prevent falls (iStoppFalls): study protocol for an international multicenter randomized controlled trial

**DOI:** 10.1186/1471-2318-14-91

**Published:** 2014-08-20

**Authors:** Yves J Gschwind, Sabine Eichberg, Hannah R Marston, Andreas Ejupi, Helios de Rosario, Michael Kroll, Mario Drobics, Janneke Annegarn, Rainer Wieching, Stephen R Lord, Konstantin Aal, Kim Delbaere

**Affiliations:** 1 Neuroscience Research Australia, University of New South Wales, Barker Street, Randwick, Sydney, New South Wales 2031, Australia; 2 Institute of Movement and Sport Gerontology, German Sport University Cologne, Am Sportpark Muengersdorf 6, 50933 Cologne, Germany; 3 Assisted Healthcare Information Technology Safety and Security Department, AIT Austrian Institute of Technology GmbH, Donau-City-Strasse 1, 1220 Vienna, Austria; 4 Institute of Biomechanics of Valencia, University Polytechnic of Valencia, Edificio 9C Camino de Vera s/n 46022, Valencia, Spain; 5 Personal Health Department, Philips Research Europe, High Tech Campus 34, 5656AE Eindhoven, The Netherlands; 6 Institute for Information Systems, University of Siegen, Hölderlinstrasse 3, 57076 Siegen, Germany

**Keywords:** Fall prevention, Fall risk assessment, Older adults, Exercise, Strength, Balance, Exergames, Video games

## Abstract

**Background:**

Falls are very common, especially in adults aged 65 years and older. Within the current international European Commission’s Seventh Framework Program (FP7) project ‘iStoppFalls’ an Information and Communication Technology (ICT) based system has been developed to regularly assess a person’s risk of falling in their own home and to deliver an individual and tailored home-based exercise and education program for fall prevention. The primary aims of iStoppFalls are to assess the feasibility and acceptability of the intervention program, and its effectiveness to improve balance, muscle strength and quality of life in older people.

**Methods/Design:**

This international, multicenter study is designed as a single-blinded, two-group randomized controlled trial. A total of 160 community-dwelling older people aged 65 years and older will be recruited in Germany (n = 60), Spain (n = 40), and Australia (n = 60) between November 2013 and May 2014. Participants in the intervention group will conduct a 16-week exercise program using the iStoppFalls system through their television set at home. Participants are encouraged to exercise for a total duration of 180 minutes per week. The training program consists of a variety of balance and strength exercises in the form of video games using exergame technology. Educational material about a healthy lifestyle will be provided to each participant. Final reassessments will be conducted after 16 weeks. The assessments include physical and cognitive tests as well as questionnaires assessing health, fear of falling, quality of life and psychosocial determinants. Falls will be followed up for six months by monthly falls calendars.

**Discussion:**

We hypothesize that the regular use of this newly developed ICT-based system for fall prevention at home is feasible for older people. By using the iStoppFalls sensor-based exercise program, older people are expected to improve in balance and strength outcomes. In addition, the exercise training may have a positive impact on quality of life by reducing the risk of falls. Taken together with expected cognitive improvements, the individual approach of the iStoppFalls program may provide an effective model for fall prevention in older people who prefer to exercise at home.

**Trial registration:**

Australian New Zealand Clinical Trials Registry Trial ID: ACTRN12614000096651.

International Standard Randomised Controlled Trial Number: ISRCTN15932647.

## Background

In the next decades a rise in the proportion of people aged 65 years and older is expected [1,2]. Successful independent living in older people can be compromised by a number of key health conditions including heart disease, stroke, diabetes, and falls [3]. About one third of community-dwelling older people falls at least once a year [4], increasing to half of people aged 80 years and over [5]. Falls can be devastating, contributing to a considerable increase in mortality and morbidity [6]. It is therefore crucial to invest in research aimed at dealing with health challenges of an ageing population such as fall prevention. The prevention of falls and its serious consequences (e.g., hip fracture) may enable older people to live independently, maintain their quality of life, and reduce health care costs [7].

Several systematic reviews and meta-analyses have provided robust evidence to support interventions for preventing falls in older people [8,9]. Exercise interventions are one of the single most effective strategies for preventing falls [10]. Systematic review evidence suggest that, to be effective in preventing falls, exercise programs must include at least moderately-challenging and progressive balance exercises and be performed frequently (i.e., for more than 50 hours over the course of the intervention period) [10]. Despite evidence of the benefits of exercising, several barriers have been identified that affect participation such as unsuitable schedules of group sessions, insufficient means of transport to get to training facilities, lack of time to exercise due to other social commitments, and feelings of loneliness and isolation [11]. More research on alternative approaches for delivery of exercise programs is needed to address the challenges and improve adherence to exercise programs, especially for older people and more socially deprived people who are at the highest risk of falling. Novel and engaging methods have great potential to enhance long-term motivation and adherence without increasing costs.

The proposed intervention will investigate the effects of an individually tailored ICT-based exercise program delivered through the home television set, called iStoppFalls ( http://www.istoppfalls.eu). The results will allow evaluation of the intervention regarding feasibility and acceptability, quality of life, as well as effectiveness on fall risk factors in older people.

## Methods/Design

### Participants

**Figure 1:**
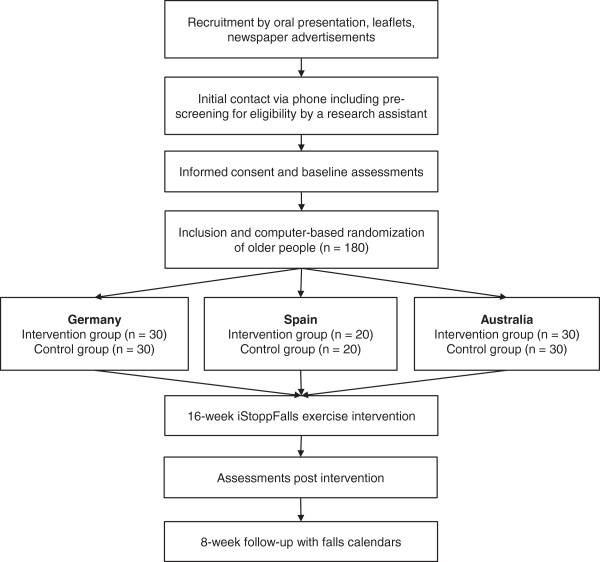
Flow chart of study design.

One hundred sixty community-dwelling older people aged 65 years and older will participate in this international, multicentre, single-blinded, two-group randomized trial (Figure 1). Study sites are located in Cologne, Germany (n = 60), Valencia, Spain (n = 40), and Sydney, Australia (n = 60). Participants will be recruited between November 2013 and May 2014. Older people will be enrolled if they meet the following eligibility criteria: (1) aged 65 years and older, (2) living in the community, (3) able to walk 20 m without a walking aid, (4) able to watch television (TV) with or without their glasses from 3 m distance, and (5) have enough space for system use (3.5 m^2^). The exclusion criteria are: (1) insufficient language skills to understand the study procedures, (2) cognitive impairment (Mini-Cog: 1–2 recalled words and abnormal clock drawing test) [12], and (3) medical conditions (i.e., uncontrolled hypertension, severe neurological disorder, acute cancer, psychiatric disorder, acute infections) that prevent participation in a regular exercise program. A medical clearance form has to be issued by the participant’s medical doctor to confirm medical suitability for the intervention program.

### Randomization and blinding

Following successful baseline assessments, participants will receive a unique computer-generated random number for identification (ID). A research assistant will then allocate the participants’ ID to the intervention or control group (ratio 1:1) by using permuted block-randomisation (software available from https://apps.neura.edu.au/blinders/). Couples (participants living in the same household) will be treated as one unit and randomised into the same block. Reassessment will be performed by experienced and trained research assistants (RA) who are blinded to group allocation. To avoid unblinding of the RA, participants will be reminded not to talk about their user experience during the reassessments.

### Study design

The iStoppFalls system consists of a technology-supported fall prevention program and a fall risk assessment. Based on discrete measuring technologies the system predicts the individual fall risk, offers a tailored and targeted exercise program, and provides individual feedback to the participant. For the iStoppFalls project, custom fall prevention software has been developed. The iStoppFalls software platform consists of new video game-based exercises and exergames focussing on balance and muscle strength, and a fall risk assessment. After randomization, the participants in the intervention group will be provided with a personal computer (Shuttle Barebone Slim-PC), a Google TV set top box (STB) by Sony, a Microsoft Kinect, a Senior Mobility Monitor (SMM) by Philips, and a Nexus 7 Android tablet.

Research staff will install the system components in participants’ homes. During the home visit of about two hours, participants will receive an introductory lesson in system use and an instruction manual. Participants will receive a follow-up home visit approximately two weeks after the installation to ensure safe use and progression of training, and to discuss any issues related to using the program. Additional home visits will be offered as needed or requested. The control group will follow their habitual exercise routine. Each participant will be provided with an educational booklet about general health and falls. Participants will be assessed at baseline (0 weeks), after 8 weeks, and at the end of the intervention period (16 weeks).

Adherence will be monitored by the iStoppFalls system which tracks the exercise activity of each participant. Falls frequency will be monitored with monthly fall diaries in control and intervention participants for 6 months after randomisation. A fall will be defined using the internationally derived consensus definition of ‘an unexpected event in which the participant comes to rest on the ground, floor, or lower level’ [13]. If the calendars are not returned, filled out incorrectly, or show non-adherence, participants will be contact by phone by a blinded (falls) and unblinded (adherence) RA, respectively. Participants will receive individual training reminders in the middle and the end of each week via the iStoppFalls system. Additionally, participants will be reminded by an unblinded RA to perform at least one interactive fall risk assessment (including questions about fall history and self-administered physical tests of balance, reaction time, and strength) each month.

Participants will be required to give written informed consent prior to inclusion. Ethical approval was obtained by the ethics committees of the German Sport University Cologne (24.09.2013), the Polytechnic University of Valencia (19.12.2013), and the Human Research Ethics Committee of the University of New South Wales (reference number HC12316, 19.12.2013). This trial will be conducted according to the ethical standards of the Helsinki Declaration.

### The iStoppFalls system

**Figure 2:**
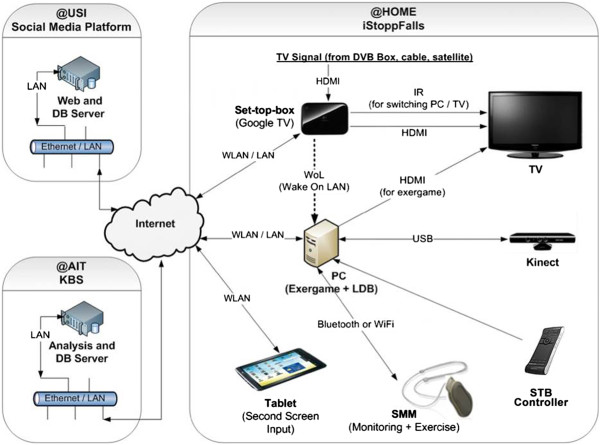
**iStoppFalls hardware system components.** AIT: Austrian Institute of Technology, DB: database, DVB: digital video broadcasting, HDMI: high definition multimedia interface, IR: infrared, KBS: knowledge based system, LAN: local area network, LDB: local database, PC: personal computer, SMM: Senior Mobility Monitor, STB: set top box, TV: television, USB: universal serial bus, USI: University of Siegen, WLAN: wireless local area network.

Participants interact with the system via the TV which is functionally extended by an android-based STB and a custom iStoppFalls application. The STB communicates with the iStoppFalls exergame software installed on the PC (Figure 2). The application was designed using an exergame approach, and will be used for the exercises and fall risk assessments. The Microsoft Kinect (3D depth sensor) and the custom-made SMM (3D accelerometer, barometer), which is worn as a necklace, will monitor the participant’s performance during the assessments and exercises. In addition, the SMM is used for continuous mobility monitoring (i.e., walking distance, sit-to-stand transfer performance) [14].

Participants control the movements of a virtual avatar with the Microsoft Kinect. The posture of the avatar is defined by the skeleton model of the Kinect Software Development Kit after applying filters to avoid jerky movements [15]. Tracked motions, joint angles, and postures will be further used to define the real-time feedback for the users who will be informed about the distance walked (in meters) equivalent to their body movements [16,17]. All aggregated data are transmitted to a knowledge-based system (server) for automatic data analysis and feedback generation. The participants will be able to monitor their results continuously via the STB over their interactive TV or tablet at home. An integrated social media platform will enable users to interact with each other and share their results.

#### Design of intervention

Following system installation, participants allocated to the intervention group will conduct a 16-week exercise program focusing on improving static balance, dynamic balance, and muscle strength for the lower extremities. The training content is based on best practice recommendations for exercise to prevent falls in older people by Sherrington et al. [10,18]. It will be recommended that the participants perform at least three balance sessions of about 40 min each (including each of the exergames) and at least three muscle strength sessions of about 15 to 20 min each (including all strength exercises) per week. Participants are recommended to perform 10 min of balance exercises before each strength training session. The weekly training duration should therefore total to about 120 min for balance training and 45 to 60 min for strength training (resulting in approximately 50 h over 16 weeks). Participants will be recommended not to do strength training sessions on consecutive days. When balance and strength training is combined, participants will be instructed to start with the balance session. The prescribed exercise intensity is moderate to high varying according to the level of difficulty, number of repetitions, and additional ankle cuff weights (1 kg or 2 kg) used. Participants in the intervention group who decide to discontinue the exercise program may still continue to send in the monthly falls calendars and perform the reassessments.

### General training principles

A sheet of ‘Exercise Safety Guidelines’ is provided to each intervention group participant. In order to maximize training effect and safety, participants are advised to hydrate well before, during, and after the exercise sessions. They should not exercise on an empty stomach or just after a big meal. It is recommended to participants to wear comfortable, well-fitted clothes, and non-slippery shoes. Participants initially start exercising at an easy level to become familiar with training and technology use. For safety reasons participants should have a chair placed to each side when exercising. Participants are advised to stop exercising and immediately consult their doctor if they feel unwell (i.e., due to a temporary illness, not taking their regular prescribed medications, having signs of angina pectoris; having difficulty breathing; and/or feeling dizzy, lightheaded or faint).

### Training content

#### Balance exercises

**Figure 3:**
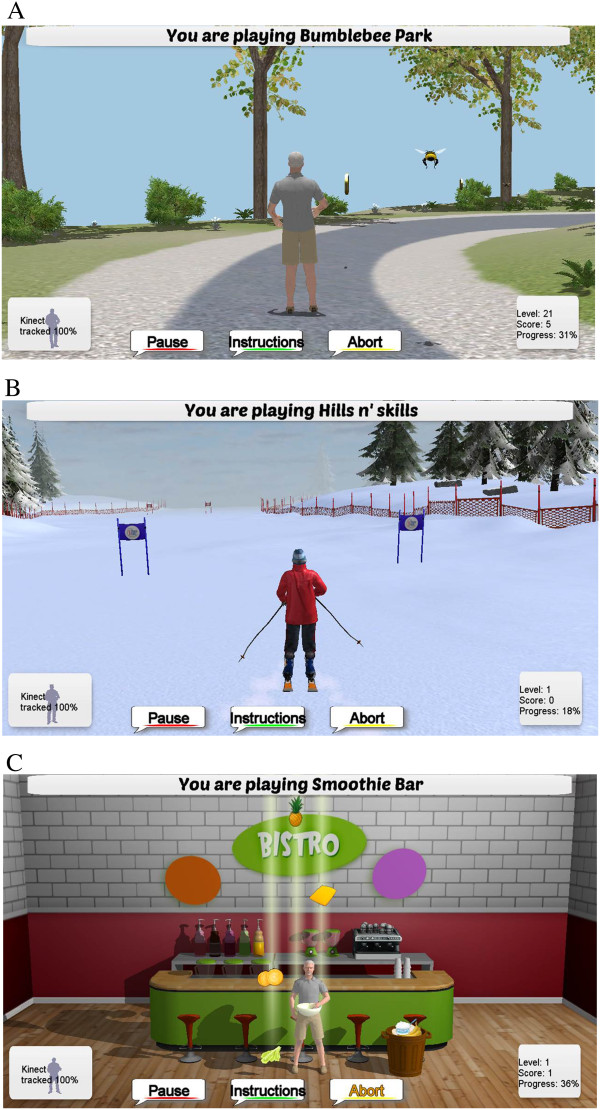
**Exergames for balance. (A)** ‘Bumble Bee Park’: walking on the spot avoiding approaching bumble bees, **(B)** ‘Hills n’Skills’: downhill skiing aiming for the gates, **(C)** ‘Balance Bistro’: stepping to the side collecting fruits.

The principles of balance exercises are based on the Otago Exercise Program [19,20] and the Weight-bearing Exercise for Better Balance (WEBB) program ( http://www.webb.org.au). The iStoppFalls program aims to improve static balance, leaning balance and stepping ability by practicing activities relevant to ADL. Three balance exergames were specifically developed for the iStoppFalls project ‘Bumble Bee Park’, ‘Hills & Skills’, and ‘Balance Bistro’ (Figure 3). All games target motor skills related to postural control including walking, weight shifting, knee bending, and/or stepping in different directions. Additionally, cognitive tasks are added once participants reach higher exergame levels (dual-tasking). In the cognitive tasks, users have to identify, memorize, and remember items, or perform mathematical calculations which randomly appear on screen. Progression of balance exergames is achieved by reducing upper limb support (from two chairs to one chair to none), narrowing the base of support, adjusting speed of movement, increasing gaming duration, and proceeding to a higher difficulty level. Proceeding to a higher level depends on a participant's high score calculated as the sum of points of the different tasks (i.e., passed gates, collected fruits, memorized items). The users will get direct (real-time) feedback about their progress by duration, score, and progress information (i.e., time, missed gates, correctly identified items) displayed on the screen.

#### Muscle strength exercises

**Figure 4:**
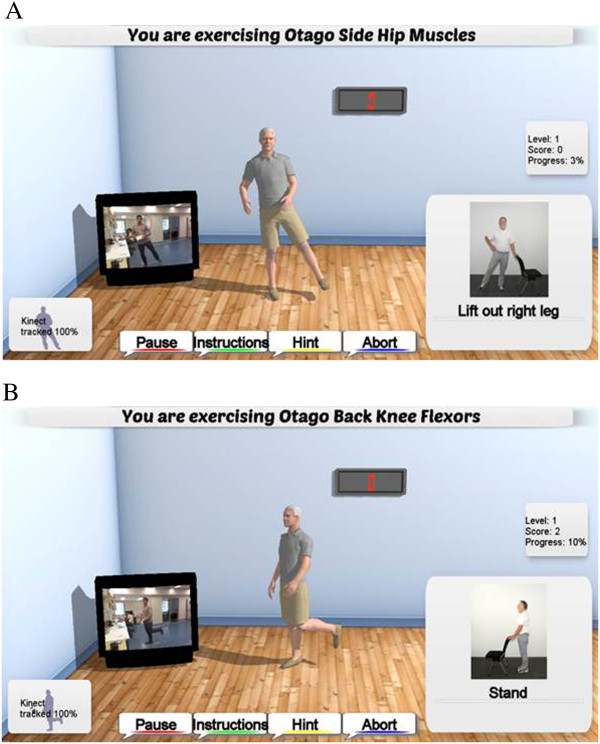
Strength exercises for (A) hip abduction muscles and (B) back knee flexor muscles.

The Ambient Assisted Exercise Program (AAEP) for iStoppFalls is based on the strength exercise component of the Otago exercise program [19]. It incorporates exercises for muscle strength of the lower extremities used in ADL and balance recovery: knee extension, knee flexion, hip abduction, calf raises, and toe raises. For muscle strength training, 2 to 3 sets of 10 to 15 repetitions and rest periods of 2 min will be recommended. To ensure technically correct movements and maximal range of motion all users will be provided with visual and verbal instructions. Progression is achieved by increasing the number of repetitions, the number of sets, and the difficulty level (e.g., by using the provided ankle cuff weights ranging from 1 kg to 3 kg). Participants will reach the next level if they correctly perform 3 sessions. Users will get direct (real-time) feedback about their progress by duration, number of repetitions, adequacy of movements, and progress information (e.g., percentage of completed task) displayed on the screen (Figure 4).

#### Education material

The educational booklet contains information about fall prevention, exercise, healthy eating, general health, footwear, medication, environmental hazards, and emergency procedures. It also includes a home safety checklist and a fall quiz. This content is based on well-known risk factors for falls, evidence-based fall prevention interventions, and guided by good practice principles [21]. The educational booklet aims to promote self-management, and offers simple and effective strategies to reduce fall risk.

#### Outcome measures

Participants will be assessed at baseline (0 weeks), in the middle (8 weeks), and at the end of the intervention period (16 weeks). The assessments will be conducted under standardized conditions at the study centres or in rooms provided by the retirement villages. Table 1 outlines the assessments performed at different time points. Each assessment will take about two hours to complete. When a stopwatch is used, time will be recorded to the nearest 0.01 s. For the Timed Up and Go (TUG), walking, and sensor-based assessments, participants will be wearing a SMM device as a necklace at the height of the sternum.

**Table 1:** Study assessments by time point

**Type of assessment**		**Outcome variable**	**Baseline assessments (week 0)**	**Re-assessments (week 8)**	**Post-assessments (week 16)**
Health	Demographics, health status, falls history	NA	X		
	12-item WHODAS 2.0	Secondary	X		X
	European Quality of Life – 5 Dimensions	Primary	X	X	X
	Iconographical – Falls Efficacy Scale	Secondary	X	X	X
	Falls and exercise adherence calendars	Secondary		Monthly	
Technical	Technology use	Secondary	X		
	SUS & PACES	Secondary		X	
	TAM & UTAUT	Secondary			X
	AttrakDiff2 & AFSS & DART	Secondary			X
Physical	Physiological Profile Assessment	Primary	X		X
	Short Physical Performance Battery	Secondary	X		X
	Sensor-based balance tests	Secondary	X	X	X
	Sensor-based reaction time tests	Secondary	X	X	X
	Sensor-based chair stand test	Secondary	X	X	X
	Timed Up and Go test	Secondary	X		X
	Steady-state walking speed (single and dual task)	Secondary	X		X
	Hand grip strength	Secondary	X		X
	IPEQ or PAQ-50+	Secondary	X	X	X
Cognition	Mini-Cog	NA	X		
	Addenbrooke’s Cognitive Examination III	Secondary	X		X
	Trail Making Test	Secondary	X		X
	Victoria Stroop test	Secondary	X		X
	Digit Symbol Coding test	Secondary	X		X
	Digit Span Backward	Secondary	X		X
	Attention Network Test	Secondary	X		X
	Counting backwards by three	Secondary	X		X
iStoppFalls system	Exercise adherence	Secondary		Continuously	
	User activity (i.e., set top box, tablet)	Secondary		Continuously	
Kinect sensor	Range of motion	Secondary	While exercising		
	21 Joint angles	Secondary	While exercising		
SMM sensor	Walking distance (m)	Secondary		Daily	
	Peak power sit-to-stand transfers (W)	Secondary		Daily	

AFSS = Activity Flow State Scale; DART = Dynamic Acceptance Model for the Re-evaluation of Technologies; IPEQ = Incidental and Planned Activity Questionnaire; NA = non-applicable; PACES = Physical Activity Enjoyment Scale; PAQ-50+ = Physical Activity Questionnaire for the population aged 50 years and older; SMM = Senior Mobility Monitor; SUS = System Usability Scale; TAM = Technology Acceptance Model; UTAUT = Theory of Acceptance and Use of Technology; WHODAS = World Health Organization Disability Assessment Schedule.

#### Demographic and general health

Participants will be sent a self-report questionnaire by post and will be requested to complete it prior to the baseline assessment. We will collect information on socio-demographic characteristics (age, gender, marital status, ethnicity, housing situation, economic status, previous occupation, education) and medical history (presence of medical conditions, medication use, history of falls). In addition, the following outcome measures will be assessed at baseline and 16 weeks follow-up.

The 12-item World Health Organization Disability Assessment Schedule (WHODAS) 2.0 is a generic assessment instrument for six domains of general health: understanding and communicating, mobility, self-care, interpersonal interactions, household and work activities, and participation in society ( http://www.who.int/classifications/icf/whodasii/en/). This self-administered questionnaire is short, simple, and easy to administer. It produces standardized disability levels and profiles linked to the International Classification of Functioning, Disability and Health (ICF) [22].

The Patient Health Questionnaire (PHQ-9) is a 9-item questionnaire for screening, diagnosing, monitoring, and measuring the severity of depression [23]. Each item is scored from 0 (not at all) to 3 (nearly every day) leading to the diagnosis of minimal (0 to 4 points), mild (5 to 9 points), moderate (10 to 14 points), moderately severe (15 to 19 points), and severe (20 to 27 points) depression. The PHQ-9 showed excellent internal reliability (Cronbach’s alpha = 0.86) and discriminant validity (area under the curve = 0.95) [23].

The European Quality of Life – 5 Dimensions (EQ-5D-5-L) questionnaire was developed to generate a basic health index ( http://www.euroqol.org/eq-5d-products/eq-5d-5l.html). Participants will describe their health status in five dimensions: mobility, self-care, usual activities, pain/discomfort, and anxiety/depression. Each dimension has three possible levels: (i) no problem, (ii) moderate problems, or (iii) extreme problems. Thus, 243 health states represented by a 5-digit combination can be defined according to the relevant level within each dimension (e.g., 11111 represents no problems on any dimension). In addition, participants will report their overall health during the past 30 days on a continuous scale (0 to 100, higher score equals better health perception) [24].

The Iconographical – Falls Efficacy Scale (Icon-FES) is an innovative way of assessing concern about falling [25]. As part of the baseline assessments, the Icon-FES will be used to investigate the participants’ concerns about falling ( http://www.neura.edu.au/apps/iconfes). Thirty pictures of daily activities, each within a specific environmental context associated with different probabilities about falling (i.e., taking the escalator, going downstairs), will be graded by the participant on a 4-point Likert scale (1 = not at all concerned, 4 = very concerned). The Icon-FES has excellent psychometric properties and shows close continuity with the Falls Efficacy Scale International (Spearman’s rho = 0.742, P < .001) [25,26].

#### Physical and functional assessments

The Physiological Profile Assessment (PPA) short version will be applied to generate an overall fall risk score based on tests which directly assess sensorimotor abilities: contrast sensitivity (Melbourne edge test (MET), peripheral sensation (proprioception), balance (sway when standing on medium-density foam with eyes open), lower extremity muscle strength (knee extension), and hand reaction time [27]. In multivariate models these variables provide an overall falls risk score, and can predict those at risk of falling with 75% accuracy in community and retirement village settings. A PPA score indicates mild (<1), moderate (1–2), and marked (≥2) fall risk in relation to a normative database. In addition, two PPA long version tests for leaning balance will be applied (coordinated stability test and maximal balance range test).

The Short Physical Performance Battery (SPPB) will be used to assess lower extremity function based on the following tests: static balance (side-by-side, semi-tandem, and tandem stance), walking speed over 4 m (at normal pace), and the five times chair stand test [28]. Each test will be assigned a score from 1 (worst) to 4 (best). Summing the category scores for the three tests can be used to create a SPPB summary performance scale.

The TUG by Podsiadlo and Richardson [29] will assess a combination of basic functionality, physical mobility, and dynamic balance [30,31]. On the word ‘go’, participants will have to stand up from a chair, walk to a three meter mark, come back, and sit down in the chair again. Time will be recorded with a stopwatch from the word ‘go’ until the participant sits down. Test-retest reliability of the TUG in older people is excellent (ICC = 0.99) [29].

Steady-state walking speed will be measured over a 10 m distance (plus 2 m for acceleration and 2 m for deceleration) by using a stop watch [32]. At the start and the finish there will be a standard chair with arm rests positioned. All walks will be performed barefoot. The participants will be instructed to walk at their comfortable normal walking speed [33]. Time is recorded with a stop watch when the participant’s limb crosses the first marker until the participant’s limb crosses the second marker. During the second walk, participants will be asked to count backwards by three starting from a random 3-digit number (dual-tasking) [34]. The potential resulting dual task interference will give an indication of the extent to which older people slow down when simultaneously walking and counting [35].

A hydraulic hand grip dynamometer (Europe: Jamar Dynamometer, Lafayette Instrument, Lafayette, IN, USA; Australia: North Coast Hydraulic Hand Dynamometer, North Coast Medical, Inc., Morgan Hill, CA, USA) will be used to assess hand grip muscle strength. Participants will sit in a comfortable chair with the elbow flexed to 90 degrees, the wrist in a neutral position, and the shoulder adducted and neutrally rotated. After the participant exerts maximal force, strength is recorded to the nearest 1 kg. Each participant has three attempts while the highest score is used for analysis. Low muscle strength will be classified as hand grip strength <30 kg in men and <20 kg in women according to the European Working Group on Sarcopenia in Older People consensus paper [31].

To assess the participants’ level of physical activity, the Incidental and Planned Activity Questionnaire (IPEQ) [36] will be used in the Australian and Spanish study arm. The IPEQ consists of a self-report questionnaire which focusses on the frequency and duration of several levels of planned and incidental physical activity in older people. This questionnaire focuses on weekly physical activity including planned exercises or walks, and more casual day-to-day activities. The total duration of physical activity is summed across all components and expressed as hours per week. Measurement properties and concurrent validity for the IPEQ have been reported to be excellent [36]. In the German study arm the Physical Activity Questionnaire for the population aged 50 years and older (PAQ-50+) [37] will be applied. It evaluates physical activity during an average week of the past month. This questionnaire estimates physical activity of older people based on exercise, housework, gardening, job, and leisure activities. By multiplying the metabolic equivalent of an activity by the duration of performing the physical activity an estimate of total energy expenditure can be obtained. The PAQ-50+ showed an acceptable test-retest reliability of 0.52 to 0.60 [37].

#### Sensor-based physical assessments

**Figure 5:**
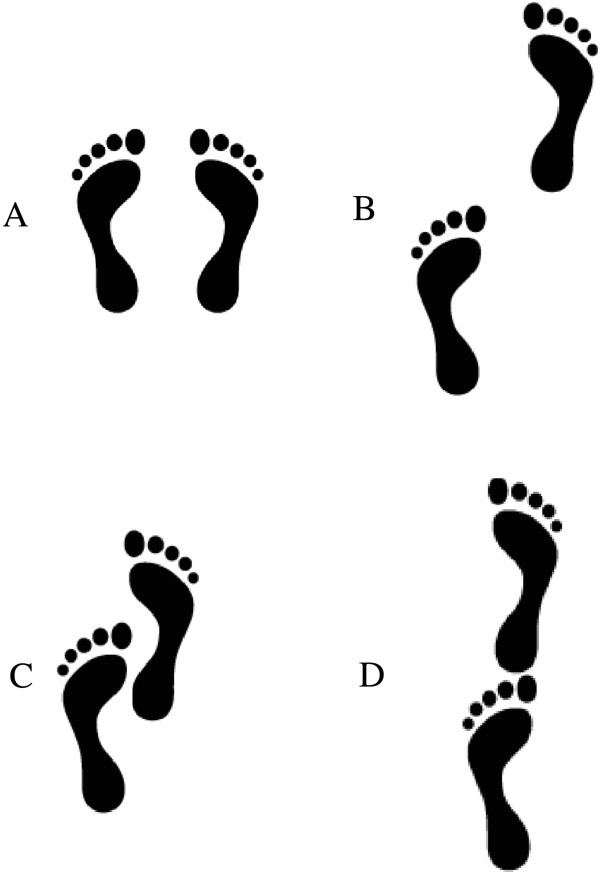
**Base of support during static balance sensor-based assessments. (A)** bipedal stance, **(B)** near-tandem stance, **(C)** semi-tandem stance, **(D)** tandem stance. Picture by courtesy of bfu - Swiss Council for Accident Prevention.

Four sensor-based physical assessments using the iStoppFalls software, the Microsoft Kinect, and the SMM will be performed barefoot in front of a TV: (1) Balance tests include comfortable-bipedal, semi-tandem, near-tandem, and tandem stance (Figure 5). Each balance task will be repeated twice for a maximum duration of 30 s with the preferred foot in front (no changes allowed between the different stances) and eyes open. After the countdown ‘ready, set, go’, time will be stopped when the participant moves his/her feet, touches the chair for support, or successfully reaches 30 s. (2) The hand reaction time test will require participants to use their hands to hit two randomly flashing lights positioned to the left and right on a virtual table. Two sets of 20 repetitions will be performed. (3) For the stepping reaction time test, two randomly flashing lights will be positioned to the left and right on a virtual floor. Participants will need to perform a lateral step onto the flashing light as fast as possible. Stepping should include weight-shifting rather than only foot tapping to the side. Two sets of 20 repetitions will be performed. (4) The sit-to-stand test is a functional measure for lower extremity strength, power, and balance. Participants will be instructed to cross their arms over the abdomen, and rise from a chair for five times as quickly as possible. Time measured by a stop watch indicates insufficient (≥16.7 s), sufficient (13.7 s to 16.6 s), good (11.2 s to 13.6 s), and very good strength performance (≤11.1 s) [38].

### User activity and adherence

Based on the real-time user activity tracking inside the exergame (biomechanical model of the Kinect sensor) and overall iStoppFalls system (SMM and all human computer interactions), this study will be able to measure adherence to the protocol and correlate these results with the assessment outcomes. This will include the datasets of all exercises for each participant including complete biomechanical analysis of the range of motion as well as the quality of exercises performed in the balance games and strength training.

#### Neuropsychological assessments

The Mini-Cog will be used as a screening tool for cognitive impairment which forms part of the exclusion criteria [12]. It consists of a 3-item recall and a clock drawing test (CDT). Participants recalling none of the words will be classified as cognitively impaired, those recalling all three words will be classified as cognitively healthy, and those with intermediate word recall (1 to 2) will be classified based on the CDT (abnormal = impaired, normal = healthy). For reasons of copyright protection, approval for the use of the Mini-Cog has been obtained by Dr Soo Borson.

The Addenbrooke’s Cognitive Examination III (ACE-III) is a brief cognitive assessment including five domains: attention, memory, verbal fluency, language, and visuospatial abilities ( http://www.neura.edu.au/frontier/research/test-downloads/). The total score is 100 with higher scores indicating better cognitive functioning. The ACE-III replaces the previous Addenbrooke’s Cognitive Examination-Revised (ACE-R) showing high sensitivity and specificity at cut-offs previously recommended: 88 (sensitivity = 1.0; specificity = 0.96) and 82 (sensitivity = 0.93; specificity = 1.0) [39]. Internal reliability using Cronbach’s alpha was good for the ACE-III (0.88) [39].

The Trail Making Test (TMT) serves as a measure of executive function (divided attention), processing/motor speed, and mental flexibility [40,41]. The participants will be asked to connect 25 encircled numbers (Part A) randomly arranged on an A4-sized paper in the correct order by using a pencil. In Part B, the participants will have to draw lines alternating between a total of 25 numbers and letters. For each part there will be a practice trial consisting of eight circles. The TMT is scored by measuring the time for the completion of each part, and by calculating ratio [Part B / Part A] and difference [Part B – Part A] scores [42].

Cognitive control will be assessed by the Victoria Stroop Test (VST) [41]. Participants will have to maintain a goal in mind and supress habitual responses to correctly perform the task. The VST consists of 24 items on each of three tasks: (1) identifying the colour of dots displayed in blue, green, red, or yellow; (2) identifying the colour of common words and disregarding their verbal content; and (3) identifying the ink colour of displayed colour words (e.g., ‘blue’ is written in red ink). A PC using the Psychology Experiment Building Language (PEBL) software version 0.13 in combination with a custom-made button device will be applied to perform the VST. The number of errors and time taken for each task will be recorded and stored locally on a PC.

The Digit Symbol Coding Test (DSC) is a multifaceted task (i.e., motor speed and incidental learning) from the Wechsler Adult Intelligence Scale (WAIS) III and will be used to investigate processing speed [43]. It consists of nine symbols that are paired with numbers. Participants will be required to copy as many symbols as possible within 120 s. The primary measure of this test is the number of correct symbols.

The Digit Span Backward (DSB) from the WAIS-III is a measure of working memory, attention, and concentration [43]. Participants will be required to repeat 2- to 9-digit numbers in the reverse order as stated by the investigator [41]. Every task consists of two sets of numbers. If the participant fails to repeat both sets of numbers, the test will be terminated. Each correctly repeated set of numbers will be scored with one point.

The Attention Network Test (ANT) will be used to quantify the processing efficiency within three attentional networks: alerting, orienting, and executive attention [44]. In this study a computer-based version of the ANT will be applied (PEBL software version 0.13) [45]. The ANT requires participants to determine whether a central arrow points to the left or right. Efficiency of the three attentional networks (alerting, orienting, and executive function) is assessed by measuring how response times are influenced by alerting cues, spatial cues, and flankers. All data will be recorded and stored locally on a PC.

A single task measure of counting backwards by three will have to be obtained to measure the amount of interference caused by simultaneously walking and counting backwards by three (dual-tasking) [35]. In this single task, participants will sit in a chair and start counting backwards by three from a random 3-digit number. The time stopped will be equal to the time used for the 10 m walk. To quantify interference, ratios between the correct number of answers during single-tasking will be compared to the number of correct answers during dual-tasking (dual task costs = [100 * (single task score – dual task score)/single task score] [46]. If the participant continues to count correctly after making a mistake, this will only be counted as one mistake.

#### Feasibility, usability, and user acceptance surveys

All participants will be required to complete a technology survey which has been adapted from an earlier survey created by H. R. Marston [47]. It includes questions on ownership of technological devices (i.e., type of computer, games console, tablet, and mobile phone), self-perceived ability to learn how to play digital games, internet use, intergenerational relationships, length of time and frequency of digital game playing, preference of digital game genres, intention to play digital games in the future, purchasing habits of digital games, purpose for buying the digital games, and social activities.

Usability and enjoyment will be assessed in the intervention group using the System Usability Scale (SUS) and the Physical Activity Enjoyment Scale (PACES). The PACES [48,49] will assess enjoyment of the exercises, and the SUS [50] will assess the usability of the iStoppFalls system.

The Activity Flow State Scale (AFSS) created by Payne et al. [51] was adapted from an earlier scale called the Flow State Scale (FSS) created by Jackson and Marsh [52]. The AFSS comprises of 26 items which are measured on a 5-point Likert scale ranging from 1 (strongly disagree) to 5 (strongly agree).

The AttrakDiff2 questionnaire initially created and further developed by Hassenzahl et al. [53,54] will be used to examine the usability of the exergames by addressing hedonic and pragmatic qualities, attractiveness, as well as identification with and stimulation through the iStoppFalls program. The questionnaire comprises 28 items which are measured on a 7-point Likert scale.

The Technology Acceptance Model (TAM) [55] is applied to measure the user acceptance of a system or technology. In its original form the TAM provides two dimensions of technology acceptance ‘perceived benefit’ and ‘perceived ease of use’. In this study an extended version as suggested by F. Davis [56] was used, including the dimensions ‘perceived effort’ and ‘perceived design’. Constructs of the Unified Theory of Acceptance and Use of Technology Model (UTAUT), as well as computer self-efficacy and trust will be added to the original TAM. The analyses will show the validity of this acceptance model in regard to the user acceptance of iStoppFalls.

#### Mid-term assessments

After eight weeks, participants will be sent the following questionnaires by mail: Icon-FES, IPEQ or PAQ-50+, and EQ-5D-5-L. For the intervention group, the sensor-based physical assessments will be repeated at the participants’ homes.

#### Statistical analyses and sample size

A priori power analysis has been conducted for PPA as the primary outcome by using data from a previous study. The estimated sample size of 52 participants for the individual study sites in Cologne and Sydney is based on a large effect (f = 0.40) for the PPA (ANCOVA, alpha 5%, power 80%, numerator df 1, number of groups 2, number of covariates 1) [57]. With an anticipated dropout of 15%, 60 participants will be recruited. The sample size will be sufficient for determining clinically significant between-group and within-group differences as well as main interaction effects in the continuously-scaled physical and cognitive outcome measures. For this study the intention-to-treat method will be used. Data on feasibility will be analysed using descriptive techniques. Student t-tests (for continuous variables with normal distribution), Fisher's exact test (for nominal data, small sample size), and Mann–Whitney U test (for ordinal or continuous data without normal distribution) will be used to determine differences between the intervention and control group at baseline. Analysis of covariates (ANCOVA) will be used to determine the intervention effect on outcome measures at follow-up adjusting for baseline values. Negative binomial regression will be used to test for differences in fall rates between groups. Paired t-tests and repeated measures ANOVA will be used to analyse changes within groups and to explore subgroups (e.g., different doses) in the intervention group. The alpha level will be set at 5%. Analyses will be performed with SPSS version 23 for Windows (SPSS, Inc., Chicago, IL). Reporting of the trial will follow CONSORT [58] statement for non-pharmacological interventions and the SPIRIT [59] guidance for protocols of clinical trials.

## Discussion

This study is designed to evaluate the feasibility and effectiveness of an easy to administer ICT-based system for fall prevention in older people living independently at home. In a large recent meta-analysis, home-based exercise has shown the potential to reduce the rate of falls (rate ratio 0.68, 95% CI 0.58 to 0.80) and fall risk (risk ratio 0.78, 95% CI 0.64 to 0.94) [8]. Previous studies showed that exercise positively affecting physical, cognitive, and functional performance comprises of a combination of balance and strength [60,61]. The iStoppFalls program has been developed to offer simple and enjoyable exercises to older people in their home environment. Particularly those older people who do not like to participate in traditional exercise programs (i.e., group training, gym workouts), are not willing to leave their house (e.g., caring for their partner), or struggle to get to the training grounds (e.g., insufficient public transport services) may benefit from exercising on the iStoppFalls system at home.

In the past decade, a wide range of computer-based and console-based videogames (i.e., Sony Playstation®, Nintendo Wii®, Microsoft Xbox®) has been developed to improve health, education, and behavioural variables [62], however, evaluated evidence-based exergames remain sparse. Virtual, interactive environments have potential to stimulate physical, cognitive, and sensory measures crucial to maintain postural control and thus prevent falls [63]. The current trial will provide data on how the iStoppFalls ICT-based exercise intervention affects balance (e.g., sway), strength (e.g., lower extremity strength), ADL (e.g., gait velocity), cognition (e.g., attention), health (e.g., quality of life), and rate of falls.

Virtual reality and gaming technology for older people have the potential to be incorporated into the clinical and home environment in the near future [64]. However, current commercially available systems are not tailored for use in older people. Our current trial will address these issues and provide data on feasibility (e.g., individual use at home), usability (e.g., navigating through a program), safety (e.g., adverse events), adherence, and enjoyment of using ICT-based systems to facilitate successful ageing. The iStoppFalls program and this trial will provide insights into older people’s attitudes and practices regarding ICT use and support of self-management of health (falls) by means of continuous monitoring of their own fall risk and associated measures like a daily activity profile, balance games, and evidence-based strength training to prevent falls. We hypothesize that ICT-systems such as iStoppFalls have the potential to provide effective means for reducing fall risk in older people.

## Competing interests

The PPA (NeuRA FallScreen) is commercially available through Neuroscience Research Australia. The Incidental and Planned Exercise Questionnaire (IPEQ) is available as a not-for-profit iPad application through Neuroscience Research Australia (NeuRA). The authors declare that they have no other competing interests. Funding sources are declared in the acknowledgements and did not influence the design or the implementation of the study.

## Authors’ contributions

Study concept and design: all authors. Drafting of the manuscript: HRM, KD, RW, SE, YJG. Critical revision of the manuscript for important intellectual content: all authors. Obtained funding: KD, RW. Administrative, technical, and material support: JA. Study supervision: KD, RW, SE. All authors read and approved the final manuscript.

## Authors’ information

Yves J Gschwind and Sabine Eichberg are joint first authors.

## Pre-publication history

The pre-publication history for this paper can be accessed here:

http://www.biomedcentral.com/1471-2318/14/91/prepub
